# Antagonistic Activity and Mode of Action of Phenazine-1-Carboxylic Acid, Produced by Marine Bacterium *Pseudomonas aeruginosa* PA31x, Against *Vibrio anguillarum In vitro* and in a Zebrafish *In vivo* Model

**DOI:** 10.3389/fmicb.2017.00289

**Published:** 2017-02-27

**Authors:** Linlin Zhang, Xueying Tian, Shan Kuang, Ge Liu, Chengsheng Zhang, Chaomin Sun

**Affiliations:** ^1^Key Laboratory of Experimental Marine Biology, Institute of Oceanology, Chinese Academy of SciencesQingdao, China; ^2^Laboratory for Marine Biology and Biotechnology, Qingdao National Laboratory for Marine Science and TechnologyQingdao, China; ^3^College of Earth Science, University of Chinese Academy of SciencesBeijing, China; ^4^Tobacco Pest Integrated Management Key Laboratory of China, Tobacco Research Institute of Chinese Academy of Agricultural SciencesQingdao, China

**Keywords:** antagonistic, phenazine-1-carboxylic acid, marine, *Pseudomonas aeruginosa*, *Vibrio anguillarum*

## Abstract

Phenazine and its derivatives are very important secondary metabolites produced from *Pseudomonas* spp. and have exhibited broad-spectrum antifungal and antibacterial activities. However, till date, there are few reports about marine derived *Pseudomonas* and its production of phenazine metabolites. In this study, we isolated a marine *Pseudomonas aeruginosa* strain PA31x which produced natural product inhibiting the growth of *Vibrio anguillarum* C312, one of the most serious bacterial pathogens in marine aquaculture. Combining high-resolution electro-spray-ionization mass spectroscopy and nuclear magnetic resonance spectroscopy analyses, the functional compound against *V. anguillarum* was demonstrated to be phenazine-1-carboxylic acid (PCA), an important phenazine derivative. Molecular studies indicated that the production of PCA by *P. aeruginosa* PA31x was determined by gene clusters *phz1* and *phz2* in its genome. Electron microscopic results showed that treatment of *V. anguillarum* with PCA developed complete lysis of bacterial cells with fragmented cytoplasm being released to the surrounding environment. Additional evidence indicated that reactive oxygen species generation preceded PCA-induced microbe and cancer cell death. Notably, treatment with PCA gave highly significant protective activities against the development of *V. anguillarum* C312 on zebrafish. Additionally, the marine derived PCA was further found to effectively inhibit the growth of agricultural pathogens, *Acidovorax citrulli* NP1 and *Phytophthora nicotianae* JM1. Taken together, this study reveals that marine *Pseudomonas* derived PCA carries antagonistic activities against both aquacultural and agricultural pathogens, which broadens the application fields of PCA.

## Introduction

There is a perpetual need for novel antibiotics to combat pathogens in the fields of public health, agriculture and aquaculture. Microorganisms are a major resource for the discovery of new drugs. The majority of microbial natural products have been isolated from terrestrial-borne microbes (Berdy, 2005), and the importance of terrestrial microbes as sources of valuable bioactive metabolites has been well established for more than half a century. However, with the fast development in marine related fields, recent trends in drug discovery emphasize that marine microorganisms are a potentially productive source of novel secondary metabolites and have great potential to increase the number of marine microbial natural products (Waters et al., 2010). Metabolically marine strains are different from their terrestrial counterparts, and thereby, they may produce unique bioactive compounds, which are not found in their terrestrial counterparts (Jensen and Fenical, 1994; Feling et al., 2003).

*Pseudomonas aeruginosa* is a ubiquitous Gram-negative bacterium having a versatile metabolic potential and great ecological and clinical significance. The geographical distribution of *P. aeruginosa* has revealed the existence of an unbiased genetic arrangement in terrestrial isolates. In contrast, there are very few reports about *P. aeruginosa* strains from marine environments. Phenazine and its derivatives are very important secondary metabolites produced from *Pseudomonas* spp. and have exhibited broad-spectrum antifungal and antibacterial activities (Lee et al., 2003; Kumar et al., 2005; Morales et al., 2010). Furthermore, it is reported that phenazine and its derivative also have the function of inhibiting cancer cells and inducing the programmed cell death in some cancer cell lines (Lee et al., 2003; McGuigan and Li, 2014). Among the phenazine compounds, phenazine-1-carboxylic acid (PCA) is confirmed an important metabolic precursor for other phenazine derivatives and also have the antifungal and antibacterial activities (Lee et al., 2003; Pierson and Pierson, 2010; Abraham et al., 2015). In China, PCA named Shenqinmycin has received a Pesticide Registration Certification from the Ministry of Agriculture, and widespreadly used against the plant pathogens in the land agriculture related fields because of its high fungicidal efficiency, low toxicity to humans and animals, environmental compatibility, and improvement of crop production (Su et al., 2010).

Up to now, the phenazine compounds and their hosts are mainly isolated from the rhizosphere of terrestrial plants, however, the same kind of compounds from marine environment and their potential applications in aquaculture are still needed to elucidate. Notably, in addition to the land agriculture, marine aquaculture plays important roles for China’s economic development. There are about 1.33 million hectares of marine cultivable areas in China, including shallow seas, mudflats and bays. However, sudden outbreak of diseases in marine aquaculture leads to high mortality and severe economic loss (Yang and Sun, 2016). Marine *Vibrio* species are associated with large-scale losses of penaeids and also cause diseases to fish (Letchumanan et al., 2015). *Vibrio anguillarum* is the causative agent of vibriosis, a deadly haemorrhagic septicaemic disease affecting various marine and fresh/brackish water fish, bivalves and crustaceans (Yu et al., 2012). To find novel antibiotics against *V. anguillarum* is urgently needed. Preetha et al. (2015) reported that brackish water derived *Pseudomonas* sp. was antagonistic to a wide range of pathogenic vibrios, which indicates that marine derived *Pseudomonas* sp. is potential to be antagonistic probiotics in aquaculture.

In this study, a marine *Pseudomonas aeruginosa* strain PA31x isolated from the sediments in China Yellow Sea showed strong inhibitory activity against *V. anguillarum* C312. The active compound was further purified and determined as PCA by HR-ESI-MS and NMR spectroscopic analyses. Finally, the possible inhibitory mechanisms of PCA acted on *V. anguillarum*, and the prevention of zebrafish embryos from the infection of *V. anguillarum* by PCA were also investigated.

## Materials and Methods

### Bacterial Strains Isolation, Identification, and Culture Conditions

The marine bacteria strains used in this study were isolated from the sediments of China Yellow Sea and cultured in marine broth 2216E (5 g/L tryptone, 1 g/L yeast extract, one liter filtered seawater, pH adjusted to 7.4–7.6) or Luria Bertani (LB) medium (10 g/L peptone, 5 g/L yeast extract, 10 g/L NaCl, pH adjusted to 7.0) (Ausubel et al., 1992), and incubated at 28**°**C. Genomic DNA was extracted from the isolate, and PCR (polymerase chain reaction) was performed to amplify the 16S rDNA gene sequence with universal primers 27F (AGAGTTTGATCCTGGCTCAG) and 1541R (AAGGAGGTGATCCACCC). The 16S rDNA gene sequence was compared with the public databases by the NCBI-BLAST program^1^. Phylogenetic tree was constructed with the MEGA version 6 with the method of maximum likelihood to determine the phylogenetic position of the strain PA31x (Tamura et al., 2013). The pathogen *V. anguillarum* C312 was cultured in 2216E or LB medium at 28°C (Yu et al., 2012).

### Antagonistic Assay of Marine Bacterial Strains against *V. anguillarum* C312

Standard agar plate bioassay was followed to test the marine bacteria for growth suppression of *V. anguillarum* (Kumar et al., 2005). Briefly, *V. anguillarum* C312 was cultivated in 2216E broth at 28 °C overnight, and the overnight culture was diluted with 2216E broth to 1 × 10^6^ cfu/mL. Thereafter, the diluted suspension was spread evenly on the surface of 2216E agar plate. After incubation for 30 min, the fresh colonies of marine bacterial strains were seeded on the plate and incubated at 28°C for 2 days. The antagonistic effects of marine bacterial strains against *V. anguillarum* C312 were checked and recorded.

### Culture Conditions for Antibiotic Production

Active compound production by marine *P. aeruginosa* PA31x was compared in different media. Four different media containing various carbon and nitrogen source were used for culture in 250 mL flasks at 28°C for 3 days with 50 mL fermentation scale. The four media include LB broth, marine 2216E broth, modified King’s medium B (glycerol 30 mL, peptone 10 g, K_2_HPO_4_ 0.5 g, MgSO_4_.7H_2_O 0.5 g in 1 L filtered seawater, pH 7.6) and modified liquid PPM medium (peptone 10 g, glycerol 10 mL, yeast extract 10 g, dissolved in 1000 ml filtered seawater) (Kumar et al., 2005). To test the active compound production of different medium, the same volume supernatant was collected and filtrated with 0.22-μm pore size filter. Then 150 μL filtrated supernatant was added into the Oxford cup which was put on the surface of LB agar medium covered with *V. anguillarum* C312 for evaluation of the antibiotic activity.

### Purification and Identification of the Anti-*V. anguillarum* Compound from *P. aeruginosa* PA31x

To obtain the active compound against *V. anguillarum* C312, marine bacterium *P. aeruginosa* PA31x was cultured on modified liquid PPM medium in 250 mL glass flasks. The purification was executed as previously described (Kumar et al., 2005). The fermentation was carried out for 2 days at 28°C on a rotary shaker at 160 rpm. The liquid culture (67 L) was centrifuged with 8,000 rpm for 10 min and the cell-free culture supernatant was collected and mixed with equal volume of ethyl acetate. The combined organic layer was concentrated under reduced pressure and 28.6 g crude extract was obtained from 67 L PPM fermentation medium. The crude extract was then mixed with slurry of silica gel (200–400 meshes) and the mixtures were loaded in the glass column (1000 mm × 30 mm), which was successively eluted with gradient from 100% dichloromethane to 100% methanol. The antibacterial activity of individual fraction was monitored by using *V*. *anguillarum* C312 as the indicator bacterium following the filter paper disk assay with minor modification (Jain and Pandey, 2016). Firstly, the LB plates spread with *V. anguillarum* C312 cell suspensions were prepared as above. Then the sterile filter paper disks (diameter 6 mm) containing eluded fractions dissolved in CH_2_Cl_2_ were put on the surface of plates and inoculated at 28 C for 2 days. Lastly, the corresponding effective antibacterial fractions were collected and dissolved in dichloromethane-methanol mixtures (9: 1) and crystallized under reduced pressure.

The antibacterial substances were subsequently analyzed by analytical HPLC system (Agilent 1260 series) with a linear gradient from 10 to 100% MeOH containing water on a C18 reversed-phase column (ZORBAX SB-C18, 5 μm, 4.6 mm × 150mm, Agilent) and collected by semi-preparative with solvent (water : MeOH = 1:1). All the HPLC grade mobile phase solvents were filtered (0.22-μm pore size) and degassed under reduced pressure before using (Lee et al., 2003). The active compound obtained from semi-preparative HPLC system was further analyzed by HR-ESI-MS and NMR. NMR spectra were recorded on a Bruker Avance-500 Hz NMR spectrometer. ^1^H NMR and ^13^C NMR spectra were measured in deuterated chloroform (CDCl_3_) at room temperature.

### Antagonistic Assay of Purified PCA against *V. anguillarum* C312

Antimicrobial activity of the PCA was evaluated by the paper filter paper disk assay as described previously (Raio et al., 2011). Briefly, the test plate with *V. anguillarum* C312 was prepared as above. The sterile filter paper disks (diameter 6 mm) containing PCA dissolved in CH_2_Cl_2_ were put on the plates for inoculation at 28°C for 2 days. The antagonistic effects of *P. aeruginosa* PA31x against *V. anguillarum* C312 were checked and recorded. The minimal inhibitory concentration (MIC) of PCA to *V. anguillarum* C312 was determined according to (Wiegand et al., 2008) with minor modification. Briefly, the OD_600_ of exponential-phase cells of *V. anguillarum* C312 was adjusted to 0.01with Mueller-Hinton broth (MHB). Thereafter, 100 μL *V. anguillarum* C312 cells suspension was transferred into the wells of 96-well microplate with different concentration of PCA. DMSO (1%, v/v) was used as solvent to dissolve PCA and served as the negative control. The microplate was incubated at 28°C for 18 h and checked under absorbance at 600 nm after incubation. In the present study, the MIC was defined as the lowest PCA concentration which inhibited *V. anguillarum* C312 or (OD_600_ < 0.05). The IC_50_ was defined as the concentration of PCA required for the half maximal inhibitory growth of pathogens. All the treatments were executed three times independently.

### Scanning Electron Microscopy (SEM)

Microbial morphology analyses by SEM were performed to investigate the morphological changes of pathogens treated with PCA. Briefly, *V. anguillarum* C312 was incubated on the 2216E as described above, and the sterile filter paper disks (diameter 10 mm) impregnated with 100 μg purified PCA was put on the medium and incubated at 28°C for 2–7 days (Kumar et al., 2005; Xu et al., 2014). The cells from the periphery of the inhibition zone and control plates were collected for SEM assay, respectively. The samples were analyzed with a SEM (Hitachi, S-3400N) operated at 5 kV.

### Transmission Electron Microscopy (TEM)

The samples for TEM were prepared as described previously with minor modification (Feng et al., 2000). Briefly, *V. anguillarum* C312 was cultivated at 28°C in liquid LB medium at 150 rpm with shaking for 16 h, then the cells were cultivated for another 12 h in the absence or presence of 5 μg/mL PCA. The culture broth was centrifuged and washed with PBS (pH 7.2–7.4). The collected cells were fixed with 2.5% glutaraldehyde. The samples treated with or without 5 μg/mL PCA were fixed as described previously (Mei et al., 2015). All the samples were analyzed with a TEM (HT7700, Hitachi, Japan) operated at 120 kV.

### Reactive Oxygen Species (ROS) Accumulation in *V. anguillarum* C312 and Human A549 Cells as Affected by PCA

The reactive oxygen species (ROS) accumulation of *V. anguillarum* C312 was measured with the fluorescent probe 2′,7′-dichlorodihydrofluorescein diacetate (DCFH-DA) as previously described (Xu et al., 2015) with minor modification as follows. In brief, *V. anguillarum* C312 was inoculated with 2216E broth for 4–6 h and the bacterial cells whose concentration reaching approximately 1 × 10^8^ cfu/mL were collected by centrifugation (5,000 rpm, 10 min, room temperature). Then the pellet was resuspended in 1 mL DCFH-DA solution (10 μM DCFH-DA in PBS buffer pH 7.4). The cell resuspensions were added into the 2 mL centrifuge tubes and incubated at 37°C for 30 min with rotation. After washed with PBS three times, 200 μL cell resuspensions were added to microplates. Thereafter, PCA dissolved in the DMSO were added into the wells with a final concentration of 0, 1, or 2 μg/mL, respectively. The PBS buffer with 3 μg/mL Rosup (a positive compound provided by the assay kit) or without any reagent (DMSO, PCA, or Rosup) was regarded as positive or negative control. The microplates were kept at 28°C, and a fluorescence microplate reader (TECAN, M1000Pro) was used to record the fluorescence values for 120 min at every 10 min with an excitation wavelength of 488 nm and an emission wavelength of 525 nm. Each treatment was represented by three replicates, and the experiment was conducted three times.

Determination of cellular ROS for A549 lung cancer cells was performed according to the method described previously (Hao et al., 2014), and the culture of A549 cells was carried out in accordance with the ethical guidelines of Chinese Academy of Sciences. The generation of intracellular ROS was detected with the probe DCFH-DA. Briefly, A549 cells treated with a final concentration of 0, 2, 10, or 50 μg/mL of PCA for 1 hour were incubated with 10 μM DCFH_2_-DA for 30 min at 37°C in a humidified atmosphere at 5% CO_2_. Then labeled cells were washed with PBS, trypsinized, and resuspended in PBS. To quantify ROS, the fluorescence intensity was measured by flow cytometry (FACS Aria II, BD Biosciences). All the experiments were carried out three times.

### Inhibitory Activities of PCA on *V. anguillarum* C312 Infection toward Zebrafish Embryos

Wild type zebrafishes were housed in mixed-sex low density tanks at 28°C on a timer-controlled 14:10 light/dark cycle. The male and female zebrafishes (3–12 months old) from the same tank were selected to generate zebrafish embryos as described (Westerfield, 1994). Ten transparent and healthy embryos were transferred into the wells of 24-well microplate containing 1 mL water and incubated at 28°C. Different concentrations of PCA (0 μg/mL, 2 μg/mL, and 3 μg/mL) dissolved in DMSO was added into per well, and 20 μL of *V. anguillarum* C312 culture adjusted to the concentration of 2 × 10^8^ cfu/mL was added into each well at the same time. The treatment containing DMSO without adding PCA and *V. anguillarum* C312 was regarded as control. The maximal level of DMSO in experimental media was 0.1% (v/v). All the embryos were incubated at 28°C, and the hatching ratio of embryos in each group was investigated. The embryos were observed with a stereo microscope (ZEISS Stemi 2000C, German) equipped with a digital camera after 48 h post-fertilization (hpf). All animal experiments were conducted in accordance with the ethical guidelines of Chinese Academy of Sciences.

### Statistical Analyses

The results were shown as the mean ± SD (standard deviations) and the statistical differences were detected with independent sample *t*-tests (^∗^*P* < 0.05; ^∗∗^*P* < 0.01). All the measurements were executed with three independent experiments.

## Results

### Anti-*V. anguillarum* Activity of Marine Bacterium PA31x

**FIGURE 1:**
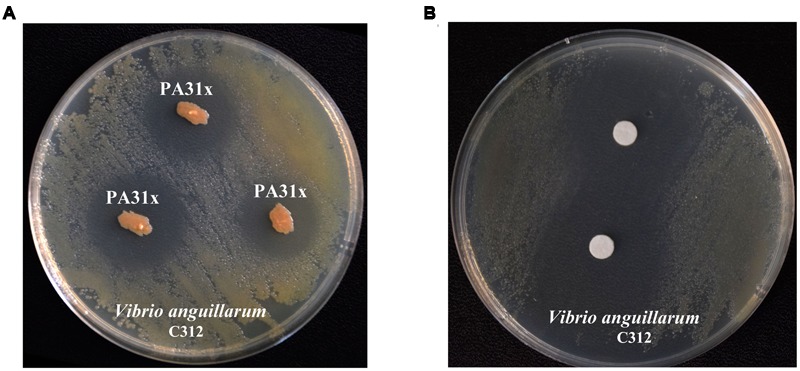
**Anti-*Vibrio anguillarum* C312 activity assays of marine bacterium PA31x (A)** and its purified active compound **(B)**. Sterile filter paper disks (diameter 10 mm) impregnated with active compound were applied in panel **B**.

In order to obtain potential anti-*V. anguillarum* compounds, over 400 bacterial strains isolated from marine sediments were evaluated by their abilities to inhibit the growth of *V. anguillarum* by antagonistic experiment. As shown in **Figure 1A**, the bacterium strain 31x in this study showed strong inhibitory activity against the growth of *V. anguillarum*. Therefore, the bacterium strain 31x was used for further study. The 16S rDNA gene of strain 31x was sequenced and aligned with other bacterial species from NCBI. Based on the alignment result, the 16S rDNA gene sequence (Accession No. KX455116) of 31x shared 99% identity with *P. aeruginosa* M18 which was isolated from terrestrial plant rhizosphere (Supplementary Figure S1). Thus, the bacterial strain 31x was identified to one member of *P. aeruginosa* and designated as PA31x.

### Purification and Characterization of Active Component from *P. aeruginosa* PA31x

To purify the active compound inhibiting growth of *V. anguillarum* from *P. aeruginosa* PA31x, the purification was performed as described in Supplementary Figure S2. The filter paper disk assay was carried out to follow the trail of active compound by using *V. anguillarum* C312 as the indicator bacterium. 26.8 g crude extract from 67 L liquid fermentation medium was mixed with same mass of silica gel and eluted. The eluted active components were further analyzed with analytical HPLC. The sole peak appeared at a retention time of 12.11 min was collected and used for anti-*V. anguillarum* assay on the 2216E plate. As expected, the purified compound showed remarkably inhibitory activity against *V. anguillarum* C312 (**Figure 1B**), which further confirmed that we successfully obtained the active component against *V. anguillarum* in *P. aeruginosa* PA31x.

### Structure Elucidation of the Antagonistic Compound

**FIGURE 2:**
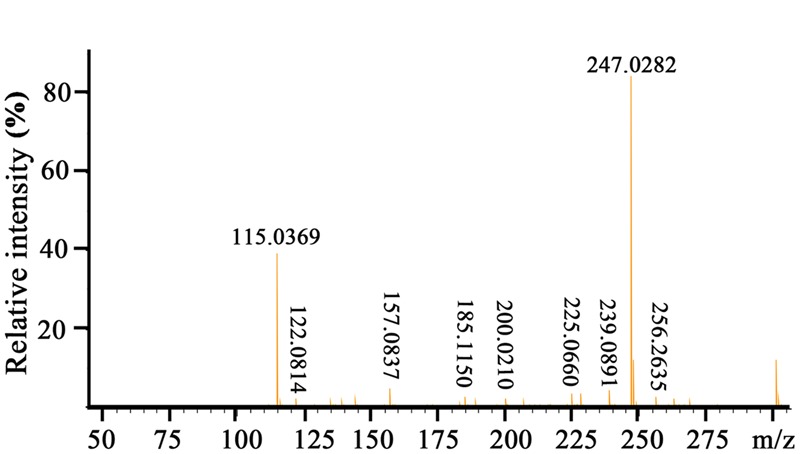
**HR-ESI-MS spectrum of the active compound produced by *Pseudomonas aeruginosa* PA31x**.

To elucidate the chemical structure of the active compound derived from *P. aeruginosa* PA31x, the HR-ESI-MS and NMR spectral data of this compound were analyzed. The HR-ESI-MS spectrum displayed a strong molecular ion peak at m/z 247.0282 (M + Na) (**Figure 2**). The GC-MS Library search result proposed the molecular formula as C_13_H_8_N_2_O_2_.

**FIGURE 3:**
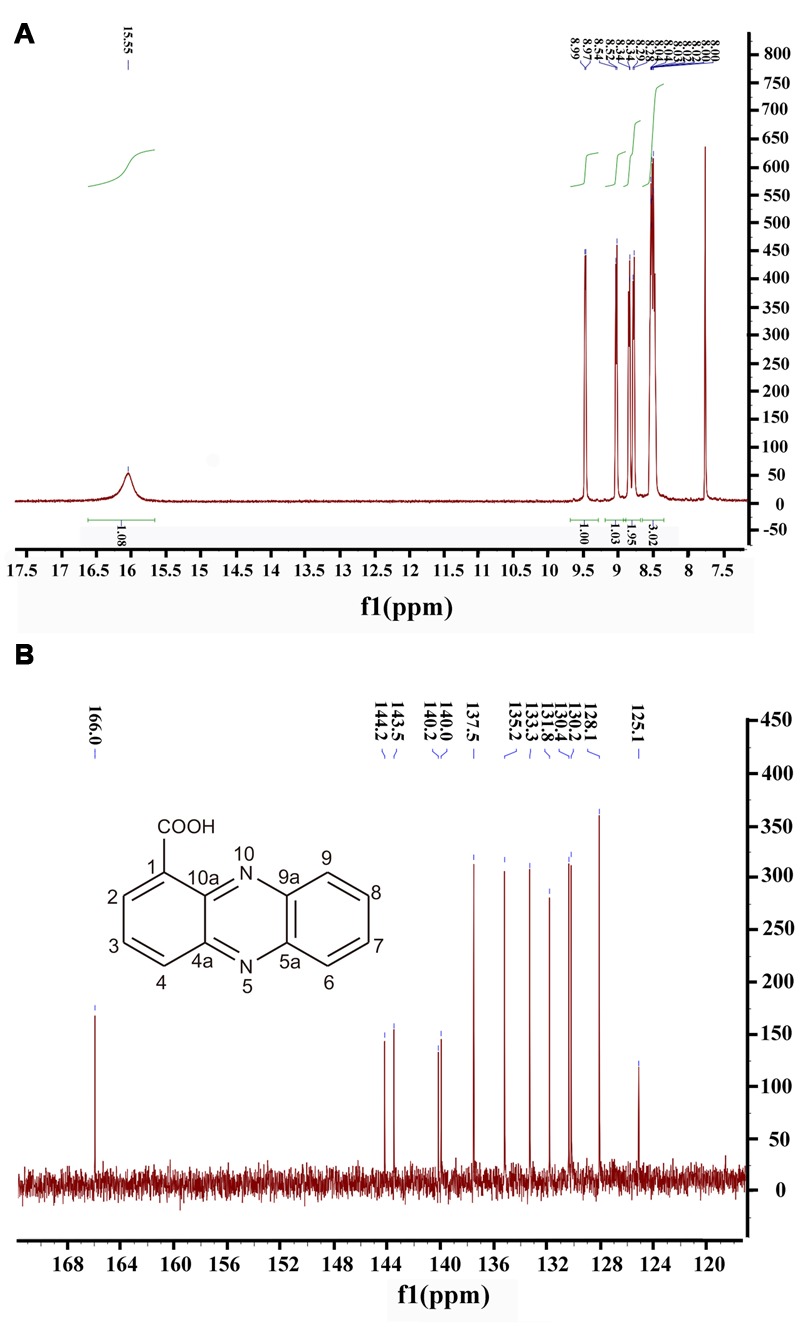
**Structure determination of active compound produced by *P. aeruginosa* PA31x by ^1^H spectral (A)** and ^13^C spectral **(B)**. The chemical structure of phenazine-1-carboxylic acid (PCA) was imbedded in the panel **B**.

Moreover, ^1^H NMR and ^13^C NMR spectroscopy were performed to clarify the exact structure of this active compound (**Figure 3**), which confirmed that eight protons (**Figure 3A**) and 13 carbons (**Figure 3B**) were present in the molecule of this compound. In the^1^H NMR spectrum, 7 methine groups and a carboxylic acid group were present at δ 8.00–8.99 and 15.55, respectively (**Figure 3A** and Supplementary Table S1). The proton at the position 2 resonated at δ 8.52–8.54. The resonance at δ 8.34 belongs to the proton at the position 3 and at the similar position in the phenazine ring, position 4 resonated at δ 8.97–8.99. Position 6 resonated at δ 8.28–8.29 and the remaining three protons at the positions 7, 8, and 9 were δ 8.00–8.04. The proton singlet at δ 15.55 was in accordance with the hydrogen-bonded carboxylic acid proton. A carbonyl group and aromatic rings at δ 166.0 and 125.1–144.2, respectively, were present in the ^13^C NMR spectrum of the active compound (**Figure 3B** and Supplementary Table S2). The resonance peak at the position 1 was δ 125.1. The resonances at δ 140.0-144.2 belong to the carbons at the positions 4a, 5a, 9a, and 10a (**Figure 3B**). The remaining resonance peaks at δ 128.1–137.5 were distributed to the carbons at positions 2, 3, 4, 6, 7, 8, and 9 of the phenazine ring (**Figure 3B**). Collectively, based on the HR-ESI-MS and NMR spectral data, the active compound derived from PA31x was characterized as PCA. In addition, antagonistic activities of PCA was dose-dependent, and the MIC of PCA for *V. anguillarum* C312 was measured. The results showed that the MIC of PCA against *V. anguillarum* C312 was 50 μg/mL. The IC_50_ of PCA against *V. anguillarum* C312 was 39.02 μg/mL.

### Cloning, Sequencing, and Alignment of Genes Involved in PCA Production

It was documented that the phenazine gene cluster *phz* had a conserved seven-gene operon, *phzABCDEFG*, which was responsible for the synthesis of PCA. Moreover, there were two nearly identical core biosynthetic gene clusters, *phz1* and *phz2*, in *P. aeruginosa* (Mavrodi et al., 2006). To investigate the genes involved in PCA production in *P. aeruginosa* PA31x, the specific primers were used to detect, clone and sequence the gene clusters, *phz1* (Supplementary Table S3) and *phz2* (Supplementary Table S4). The results showed that two gene clusters, *phz1* and *phz2*, existed in the genome of *P. aeruginosa* PA31x (**Table 1**). To further disclose the conservation of genes involved in PCA production between marine and terrestrial derived *Pseudomonas*, gene clusters *phz1* and *phz2* from *P. aeruginosa* PA31x were compared with those of *P. aeruginosa* M18 (Su et al., 2010), the typical terrestrial bacterium producing PCA. The alignment results showed that the gene clusters in *P. aeruginosa* PA31x shared high homology with those in *P. aeruginosa* M18. The identity ratio of different gene in *phz1* varied from 98.23 to 100% (**Table 1**), and the identity ratio of different gene in *phz2* varied from 98.83 to 100% when compared with those genes in *P. aeruginosa* M18 (**Table 1**).

**Table 1:** The identity comparison of antibiotic genes related to PCA biosynthesis from *Pseudomonas aeruginosa* PA31x and *P. aeruginosa* M18.

Genes	Identity (%)	Genes	Identity (%)	Genes	Identity (%)
*phzA1*	100	*phzA2*	99.80	*phzM*	99.50
*phzB1*	98.23	*phzB2*	100	*phzS*	99.67
*phzC1*	99.58	*phzC2*	99.56	*phzH*	99.45
*phzD1*	99.73	*phzD2*	99.36		
*phzE1*	99.73	*phzE2*	98.83		
*phzF1*	99.40	*phzF2*	99.16		
*phzG1*	99.38	*phzG2*	99.41		

Additionally, PCA was an important precursor which could be converted into other phenazine active compound, like PCN and PYO, with some other phenazine genes (Kerr et al., 1999). The gene *phzH* was considered to be involved in the conversion of PCA to PCN and the proteins encoded by genes *phzS* and *phzM* could transfer PCA into PYO (Kerr et al., 1999). Correspondingly, these three genes, *phzS, phzM* and *phzH* which could transform PCA to antibiotics PYO and PCN, were also detected in *P. aeruginosa* PA31x with the primers showed in Supplementary Table S5. The sequence of these three genes including *phzM, phzH*, and *phzS* were highly homologous to the phenazine genes from *P. aeruginosa* M18, and the identity ratio varied from 99.45 to 99.67% (**Table 1**). Thus, the genes involved in the production and modification of PCA are highly homologous between terrestrial and marine derived *Pseudomonas* sp..

### Morphological Changes of *V. anguillarum* C312 Cells Treated with PCA

**FIGURE 4:**
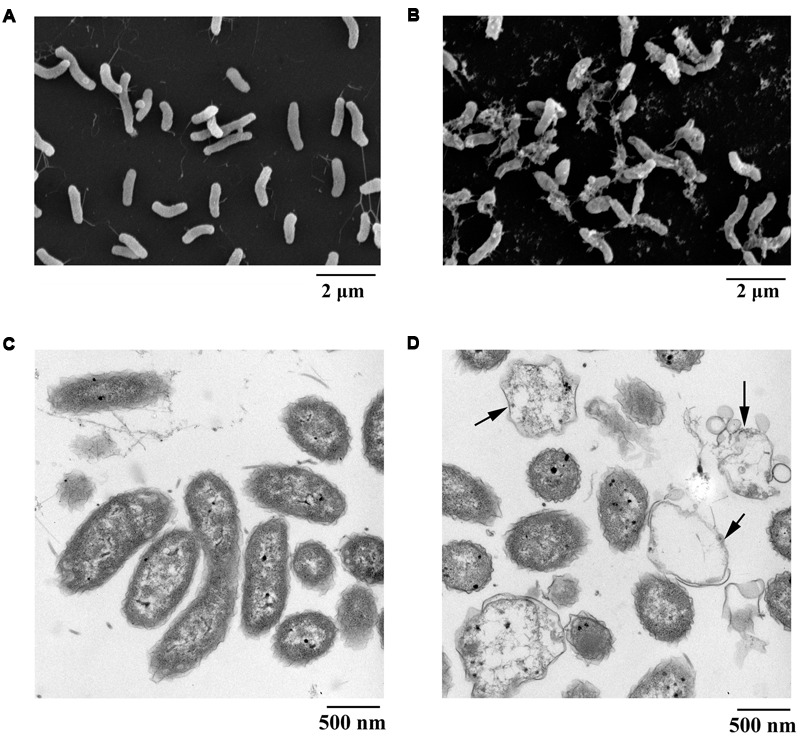
**Electron microscopic observation of morphological changes of *V. anguillarum* C312 cells following the treatment of PCA.** SEM images of *V. anguillarum* C312 cells without **(A)** or with the treatment of PCA **(B)**. TEM images of *V. anguillarum* C312 cells without **(C)** or with the treatment of PCA **(D)**.

Scanning electron microscopy is recognized as an attractive and powerful method to determine morphological changes in biological samples owing to its high-resolution imaging capability. SEM can be used to demonstrate significant alterations in cell morphology after treatment with PCA. In the present study, the pathogen *V. anguillarum* C312 was treated with the purified PCA and thereafter checked by SEM to investigate how does PCA act on the bacterium. Morphological changes of *V. anguillarum* C312 treated with PCA or DMSO were shown in **Figures 4A,B**. The surfaces of the cells in the control group were smooth and the bacteria cells were plump and round. On the contrary, the cell membranes became coarse and emerged one to several cystic bulges, and distorted with deeper grooves and corrugations after treatment with PCA (**Figures 4A,B**).

By using a transmission electron microscope, morphological material changes in the pathogenic cells following PCA treatment were further determined. It is believed that structures internal or external to the cells were altered following PCA treatment, since this treatment caused cells death. The cells of *V. anguillarum* C312 in the control group were rod-shaped with a densely-stained cytoplasm, and they were uniformly distributed (**Figure 4C**). On the contrary, the cells in the treatment group were highly variable in structure and almost devoid of nucleic acids. In addition, some cells were split open after treatment with PCA, as evident in **Figure 4D**, which indicate the presence of internal cell contents and the leakage and damage of cytoplasm. It can be concluded that the cytoplasmic content of the cells was affected and at least partially lost in the presence of PCA. The TEM images suggest that the mechanism of bacterial inactivation is mainly via the decomposition of cytoplasm. The degree of damage depends mainly on the concentration of PCA that penetrates the cell.

### ROS Accumulation in *V. anguillarum* and Human A549 Cells Treated with PCA

**FIGURE 5:**
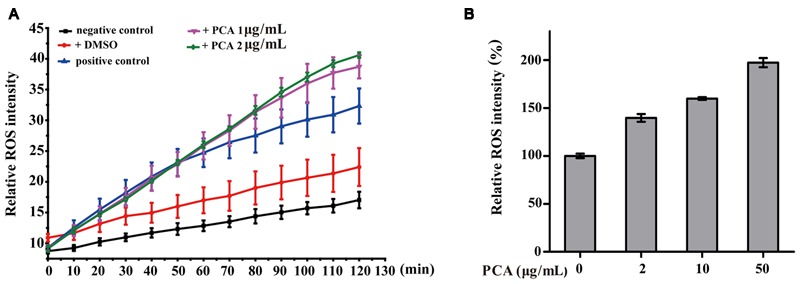
**Reactive oxygen species (ROS) accumulation in *V. anguillarum* C312 (A)** and A549 lung cancer cells **(B)** as affected by PCA. For the assay in panel **A**, the treatments included PCA in DMSO, DMSO alone (a negative control), Rosup (positive control), or none of these agents (a negative control). Values are means and standard deviations from three independent experiments.

It is known that intracellular ROS are generated in cellular response to exogenous sources such as xenobiotics compounds and pathogen invasion as well as during mitochondrial oxidative metabolism (Ray et al., 2012). To examine the intracellular ROS change by PCA, *V. anguillarum* C312 and A549 cells were treated with PCA, and ROS levels were measured by the cell-permeable substrate DCFH-DA. As evident from **Figure 5A**, the intracellular ROS accumulation in *V. anguillarum* C312 was obviously affected by PCA. It was shown that ROS product differed in the presence of PCA (0–2 μg/mL) and there was a significant increase in the intracellular fluorescence compared to the control, indicating a higher ROS production in the group treated with PCA. As a contrast, the accumulation of ROS in the negative control or DMSO treatment was much lower than those of treated with PCA or Rosup, which indicated that PCA could generate ROS in *V. anguillarum*. On the other hand, it was reported that PCA showed an antitumor activity in prostate cancer cells by ROS production and mitochondrial-related apoptotic pathway (Karuppiah et al., 2016). To examine the ROS generation by PCA in human cancer cell line, A549 cells treated with PCA were incubated with DCFH-DA. As illustrated in **Figure 5B**, A549 cells treated with PCA showed comparatively higher ROS generation than control cells. Altogether, we concluded that PCA could induce the accumulation of ROS both in *V. anguillarum* and human A549 cells.

### Inhibitory Activities of PCA on *V. anguillarum* Infection to Zebrafish Embryos

**FIGURE 6:**
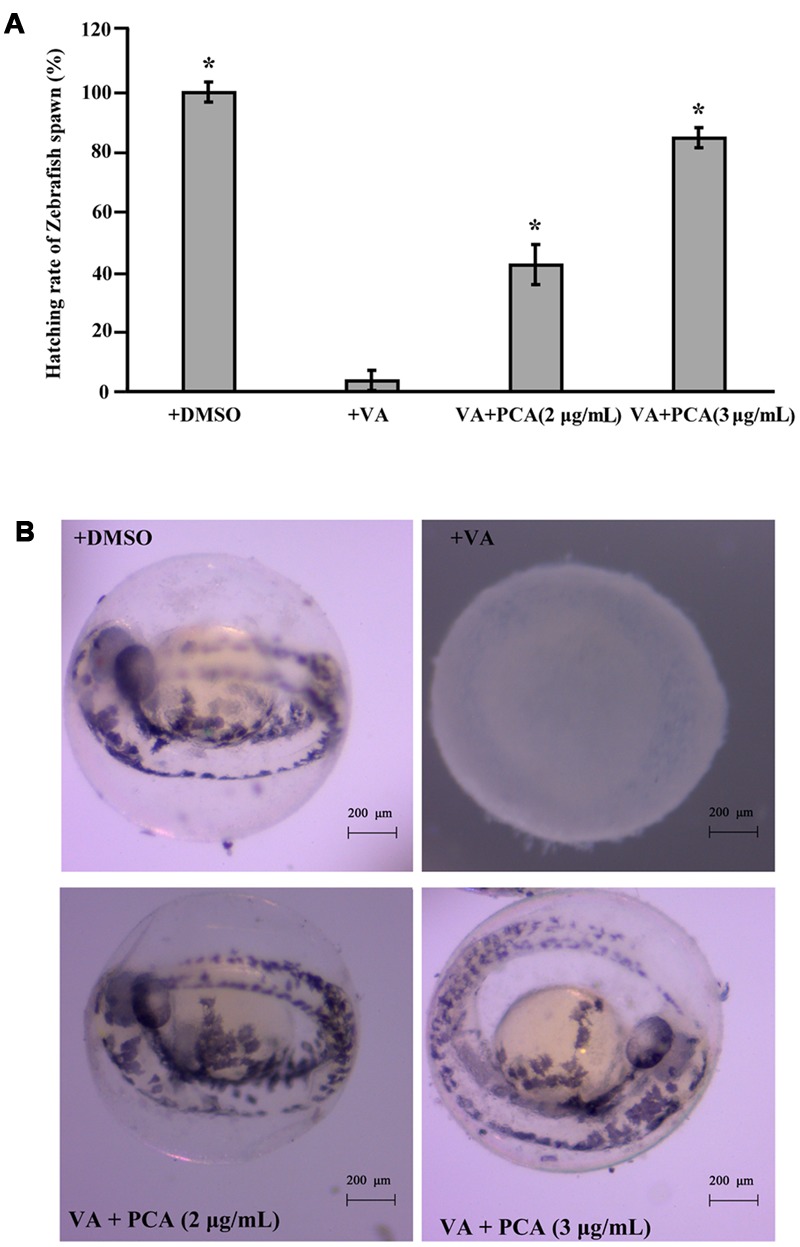
**Inhibitory activities of PCA against *V. anguillarum* C312 infection to zebrafish embryos. (A)** PCA inhibits *V. anguillarum* C312 infection to zebrafish embryos. Zebrafish embryos were infected with *V. anguillarum* C312 in the absence or presence of different concentrations of PCA and the relative hatching percent of zebrafish embryos was calculated after 48 h infection. Error bars indicate the standard deviations of three measurements. ^∗^*P* < 0.05. The error bars (SD) from the mean for three replicates are shown. **(B)** The typical hatching pictures of zebrafish embryos infected with *V. anguillarum* C312 in the absence or presence of different concentrations of PCA. All the pictures were taken with stereo microscope (ZEISS Stemi 2000C, German).

Based on our above results, PCA could effectively inhibit the growth of *V. anguillarum*. To investigate whether PCA could be applied as antibiotics in aquaculture, zebrafish was used as a model for studying the infection prevention from *V. anguillarum*. The protective assay of PCA for zebrafish embryos was performed and the hatching percent of each group embryos was calculated. As shown in **Figure 6A**, the hatching percent of embryos infected with *V. anguillarum* C312 was lowest in the groups, which was only 3.8%. However, the hatching percent of zebrafish embryos apparently raised to 42.3 or 84.62%, respectively, when 2 μg/mL and 3 μg/mL of PCA was co-incubated with *V. anguillarum* C312. With the observation of microscope, the zebrafish embryos treated with DMSO and PCA could develop normally after 48 h post-fertilization (hpf) (**Figure 6B**). However, the zebrafish embryos infected with *V. anguillarum* C312 developed abnormally and couldn’t grow to mature form (**Figure 6B**). Together, it was concluded that PCA could weaken *V. anguillarum* infection to zebrafish embryos in the water environment and would be applied as a protective agent in the aquacultural production.

## Discussion

It is reported that over 120 of the most important medicines in use today are obtained from terrestrial microorganisms. Meanwhile, the marine environment has been a source of more than 20,000 inspirational natural products discovered over the past 50 years. From these efforts, more than 22,000 discrete marine metabolites have been isolated and structurally characterized, and a significant percentage has been evaluated for some level of biological activity, such as antimicrobial effects or mammalian cell toxicity (Gerwick and Fenner, 2013). Therefore, many exciting discoveries await a more comprehensive biological evaluation of these known compounds as well as those still left to discover.

Phenazines are bacterial secondary metabolites that have long been recognized for their broad-spectrum antibiotic activity and been widely used in the biological control of arrange of bacterial and fungal pathogens (Pierson and Pierson, 2010). So far, phenazine compounds are mainly isolated from terrestrial *Pseudomonas* spp. and *Streptomyces* sp., and the marine microbe derived phenazine compounds are still rarely reported. In the present study, in a search program for microorganisms producing bioactive antimicrobial secondary metabolites effective on aquaculture diseases, the bacterial strain PA31x, which showed strong *in vitro* antibacterial and antifungal activities against some aquaculture (**Figure 1**) and plant pathogens (Supplementary Figure S3), was isolated from sediments of China Yellow Sea. The morphological, biochemical and physiological characteristics confirmed that the strain belongs to *Pseudomonas aeruginosa*. The active compound derived from *P. aeruginosa* PA31x was finally identified as PCA combining the analyses of HR-ESI-MS and NMR spectrum (**Figures 2**, **3**). As noted earlier, the antibiotic PCA belongs to the phenazine family of compounds, which have been reported to be effective against many fungi and Gram-positive bacteria but only against very few Gram-negative bacteria (Xu et al., 2015). The marine microbe derived PCA showed significant antagonistic activity against the Gram-negative bacterium *V. anguillarum*, which is consistent with the report that marine *Pseudomonas* sp. could be used as probiotics in aquaculture against *V. harveyi* (Preetha et al., 2015).

It is well known that *V. anguillarum* could cause great economic losses in the marine culture industry worldwide (Toranzo et al., 1996). More than 50 fresh and salt-water species, including some important economic species of the aquaculture industry, could be infected by *V. anguillarum* (Frans et al., 2011). To control the serious vibriosis, one of the solutions is to look for novel antimicrobial substances from natural metabolites which inhibit *V. anguillarum*. Based on our results, PCA could effectively prevent the zebrafish from *V. anguillarum* infection (**Figure 6**). Therefore, PCA would be also used as antibiotics in aquaculture fields because of its low toxicity to humans, and environmental compatibility (Yuan et al., 2008). Notably, the marine microbe derived actinonin was reported to effectively inhibit the growth of *V. anguillarum* (Yang and Sun, 2016), which indicates that it might be a good idea to try the aquatic diseases control with the reported terrestrial derived antibiotics.

Phenazine compounds have been recognized for a wide range of antimicrobial for plant pathogens and induce systemic resistance of plants (Audenaert et al., 2002; Bloemberg and Lugtenberg, 2003). Among the phenazine derivants, PCA had been used as biofungicide reagent against various phytopathogens in China (Zhou et al., 2010). In this study, marine microbial derived PCA showed significant antagonistic activity against terrestrial pathogens *Acidovorax citrulli* NP1 and *Phytophthora nicotianae* JM1 (Supplementary Figures S3–S6). *A. citrulli* causes serious seedling blight and bacterial fruit blotch of cucurbits (Bahar et al., 2011). *Phytophthora nicotianae* is a common plant pathogenic fungus infecting many important plants including tobacco, onion and strawberries (Han et al., 2016). Altogether, our results indicate that PCA has potentials to be as a biopesticide applied in both agricultural and aquacultural fields. Recently, scientists used a combined method involving gene, promoter, and protein engineering to modify the central biosynthetic and secondary metabolic pathways in the PCA-producing *Pseudomonas aeruginosa* strain PA1201 (Jin et al., 2015). The PCA yield of the resulting strain PA-IV was increased 54.6-fold and could produce 9882 mg/L PCA in fed-batch fermentation (Jin et al., 2015). Therefore, PCA has enormous potential to be applied in the aquaculture fields.

Understanding the biological effects of PCA on pathogens is essential to develop corresponding antibiotics. In the present study, on the one hand, morphologic change observed by SEM or TEM was used for studying the action mechanisms of PCA against *V. anguillarum*. It is noted that the membrane surface of *V. anguillarum* C312 emerged one to multiple protrusion of vesicles, however, the cellular contents of *A. citrulli* NP1 were released to the environment and only shaft substances of the cell were left after PCA treatment (Supplementary Figure S4), which indicated that the action mechanisms of PCA on *V. anguillarum* C312 and *A. citrulli* NP1 might be different. Additionally, PCA showed effective disease control on tobacco infected with *Phytophthora nicotianae* JM1 (Supplementary Figure S6) via distorting the mycelia of *Phytophthora nicotianae* JM1 (Supplementary Figure S5), which is consistent with the report that deformed mycelia of *Macrophomina phaseolina* appeared when this fungus was treated by phenazine compounds (Kumar et al., 2005). On the other hand, PCA is a redox-cycling agent that can be reduced both *in vitro* and *in vivo*. The reduced form PCA reacts with O_2_ and generates ROS, which would damage the cell (Xu et al., 2015). In this study, substantial ROS was accumulated in *V. anguillarum* when treated with PCA (**Figure 5A**). This result indicated that marine derived PCA, like other phenazine compounds, generates ROS in *V. anguillarum* and that ROS generation is likely to be an important mechanism of PCA action, as is the case with PCA acts on *Xanthomonas oryzae* (Xu et al., 2015).

In general, the production of specific antibiotics *in vitro* is closely correlated with their antagonism and disease control efficacy *in vivo* (Lee et al., 2003), although discrepancies exist between *in vitro* antagonistic effect and the corresponding *in vivo* efficacy in some cases. In the present study, we ascertained not only the *in vitro* antibacterial and antifungal activities of PCA against some aquaculture and plant pathogens, but also its *in vivo* antibacterial and antifungal activities in the host animal or plants. Further studies of the effectiveness of PCA for diseases control and the design of practical applications should be carried out under field conditions. Additionally, recent isolation of *P. aeruginosa* strains from an open-ocean site has shown that they possess a unique genotype (Nonaka et al., 2010), which suggests that the geographical origin of the strains is reflected in their phylogeny. Thus, it will be very interesting to investigate that the biosynthesis differences between the marine and terrestrial derived PCA in the future.

## Ethics Statement

For the human cell experiments, this study was carried out in accordance with the recommendations of ethical guidelines of Chinese Academy of Sciences with written informed consent from all subjects. All subjects gave written informed consent in accordance with the Declaration of Helsinki. The protocol was approved by the Chinese Academy of Sciences. For the zebrafish experiments, this study was carried out in accordance with the recommendations of ethical guidelines of Chinese Academy of Sciences. The protocol was approved by the ethical guidelines of Chinese Academy of Sciences.

## Author Contributions

LZ, CZ, and CS conceived and designed the experiments. LZ performed most of the experiments. XT finished the activity assay of PCA in greenhouse. SK did the ROS accumulation assay of PCA in A549 cells. GL helped to do antagonistic assay of marine bacterial strains. LZ, CZ, and CS analyzed the data. LZ and CS prepared the figures and wrote the paper. All authors reviewed the manuscript.

## Conflict of Interest Statement

The authors declare that the research was conducted in the absence of any commercial or financial relationships that could be construed as a potential conflict of interest.

## Footnotes

1 http://www.ncbi.nlm.nih.gov/BLAST
