# Supplementary material for: Antagonistic Activity and Mode of Action of Phenazine-1-Carboxylic Acid, Produced by Marine Bacterium Pseudomonas aeruginosa PA31x, Against Vibrio anguillarum In vitro and in a Zebrafish In vivo Model
Source: Front Microbiol. 2017 Feb 27;8:289. doi: 10.3389/fmicb.2017.00289 (PMC5326748; doi:10.3389/fmicb.2017.00289)
Supplement: Supplementary file 1 [file Data_Sheet_1.DOCX]

**Antagonistic activity and mode of action of phenazine-1-carboxylic acid, produced by marine bacterium *Pseudomonas aeruginosa* PA31x, against *Vibrio anguillarum in vitro* and in a Zebrafish *in vivo* model**

*Linlin Zhang^1,2,3^, Xueying Tian^4^, Shan Kuang^1,2^, Ge Liu^1,2,3^, Chengsheng Zhang^4*^, Chaomin Sun^1,2*^*

*^1^Key Laboratory of Experimental Marine Biology, Institute of Oceanology, Chinese Academy of Sciences, Qingdao, China*

*^2^Laboratory for Marine Biology and Biotechnology, Qingdao National Laboratory for Marine Science and Technology, Qingdao, China*

*^3^University of Chinese Academy of Sciences, Beijing, China*

*^4^Tobacco Pest Integrated Management Key Laboratory of China, Tobacco Research Institute of Chinese Academy of Agricultural Sciences, Qingdao, China.*

**Address correspondence to*

*Chaomin Sun*

*E-mail address:* [*sunchaomin@qdio.ac.cn*](mailto:sunchaomin@qdio.ac.cn)

*Chengsheng Zhang*

*E-mail address: zhangchengsheng@caas.cn*

**Keywords**: antagonistic, phenazine-1-carboxylic acid, marine, *Pseudomonas*, pathogens

**Running title**: Antagonistic activities of marine derived PCA

**Material and Methods**

**Bacterial Strains Isolation, Identification and Culture Conditions**

Plant pathogen *A*. *citrulli* NP1 was isolated from tobacco leaf in Nanping, Fujian, China in 2012. Plant pathogen *P. nicotianae* JM1 was isolated from tobacco stem in Jimo, Qingdao, China in 2011. *A*. *citrulli* NP1was cultured in LB medium at 28 **^◦^**C. *P. nicotianae* JM1 was cultured in oats agar medium (OA) at 28 **^◦^**C. The preparation of oat medium was performed as described previously (Han et al., 2016).

**Antagonistic Assay of Purified PCA against *A*. *citrulli* NP1 and *P. nicotianae* JM1**

Anti-*A*. *citrulli* NP1 activity of the PCA was evaluated by the paper filter paper disc assay as the same to that *V. anguillarum* C312. For the determination of antifungal activity, truffles (diameter 10 mm) taken from the edge of *P. nicotianae* JM1 cultures were placed at the center of plates. Sterile filter paper discs (diameter 10 mm) impregnated with PCA (the final concentration was 10 mg/mL) were applied at 1-3 cm from the plug margin of the culture plates. The plates inoculated with fungus alone were treated as control. The plates with or without PCA treatment were incubated at 28 **^◦^**C for 3-7 days. The antagonistic effects of *P. aeruginosa* PA31x against *A. citrulli* NP1 and *P. nicotianae* JM1 were checked and recorded. The minimal inhibitory concentration (MIC) and IC50 of PCA to *A*. *citrulli* NP1 was done as the same to that of *V. anguillarum* C312. The minimal fungicidal concentration (MFC) experiment was assessed according to the procedures described previously (Abraham et al., 2015). Briefly, same amount of fresh *P. nicotianae* JM1 culture was placed at the center of plates with different concentrations of PCA or DMSO, respectively, and incubated at 28 **^◦^**C for 2-7 days. Thereafter, the radial growth of *P. nicotianae* JM1 was measured and the corresponding of MFC was determined. All the experiments were carried out three times independently.

**Electron Microscopic Observation**

Microbial morphology analyses by SEM and TEM for *A*. *citrulli* NP1 and *P. nicotianae* JM1 were performed similar to those of *V. anguillarum* C312.

**Biocontrol Assay of PCA against *P. nicotianae* JM1 in Greenhouse**

To determine the biocontrol potential of PCA against *P. nicotianae* JM1, the healthy tobacco plants having six true leaves were chosen for pot experiments. The inoculation and spore suspensions of *P. nicotianae* JM1 for tobacco plants irrigation were performed as described previously (Han et al., 2016). Two different concentrations of solvents containing PCA (20 μg/mL and 40 μg/mL) were used to irrigate the roots of tobacco plants for three days before plants exposed to *P. nicotianae* JM1. Blank control treatment was irrigated with the same volume water containing 0.4% (v/v) DMSO. The irrigation volume for each plant was 10 mL per day. All experiments were performed three times in each group containing 15 plants. The disease severity was calculated according to the methods described previously (Han et al., 2016) and recorded on the 3rd and 7th day after pathogen inoculation.

**RESULTS**

**Inhibitory Activities of PCA on Plant Pathogens *A.citrulli* NP1 and *P. nicotianae* JM1**

It is known that PCA inhibits many microorganisms’ growth including various plant pathogens and has potential to be as a good biopesticide because of its low toxicity to humans, and environmental compatibility (Yuan et al., 2008). In the present study, we checked the antagonistic activities of marine microbial derived PCA against terrestrial pathogens *A.citrulli* NP1 and *P. nicotianae* JM1. *A. citrulli* NP1 is a Gram-negative, biotrophic bacterium and causes seedling blight and bacterial fruit blotch of cucurbits (Bahar et al., 2011). *P. nicotianae*, commonly referred to as black shank, is a plant pathogenic fungus infecting many hosts including tobacco, onion and strawberries (Han et al., 2016).  To evaluate the antagonistic activities of PCA against *A. citrulli* and *P. nicotianae*, we analyzed its antimicrobial effects by filter paper disc assay. The results showed that PCA could effectively inhibit the growth of Gram-negative bacterium *A. citrulli* NP1, which is similar to that of *V. anguillarum* C312 (**Figure S3A**). Moreover, PCA almost completely inhibited the radial mycelial growth of *P. nicotianae* JM1 (**Figure S3B**), which shows significantly antifungal activity and is consistent with the previous report (Jain and Pandey, 2016). In addition, the MIC of PCA against *A. citrulli* NP1 was 200 μg/mL. The IC_50_ of PCA against *A. citrulli* NP1 was 140.9 μg/mL. The MFC of PCA for *P. nicotianae* JM1 was 40 μg/mL.

Electron microscopic observation showed that the whole cell of *A. citrulli* NP1 was broken and the protoplast was released when treated with PCA (**Figures S4**). For *P. nicotianae* JM1, compared with the control, the hyphae became shrunken and the entocytes were released, which led to the cells death of pathogen (**Figures S5**).

Considering *P. nicotianae* is an important agricultural pathogen which could cause serious disease in many crops including tomato and tobacco. The *in vivo* efficacy of PCA for the control of *P. nicotianae* in tobacco plants was evaluated in the greenhouse. The disease severity index was recorded in the third and seventh day, after the plant exposed to *P. nicotianae* JM1, respectively. As shown in **Figure S6A**, tobacco disease indexes markedly dropped when treated with 20 μg/mL or 40 μg/ml PCA. During 7 days observation, symptoms began to appear on tobacco plants 3 days after inoculation. In the control group, plants brownish lesions occurred on the tobacco stem and extended rapidly into the upper part of plants, accompanied by a wilt of the entire plants, leaf defoliation and damping off (**Figure S6B)**. As the concentration of PCA increased, the *P. nicotianae* JM1 symptoms were progressively inhibited and eventually completely suppressed by 40 μg/mL of PCA (**Figure S6B)**.

**REFERENCES**

Abraham, A., Philip, S., Jacob, M.K., Narayanan, S.P., Jacob, C.K., and Kochupurackal, J. (2015). Phenazine-1-carboxylic acid mediated anti-oomycete activity of the endophytic *Alcaligenes s*p. EIL-2 against *Phytophthora meadii*. *Microbiol. Res.* 170**,** 229-234. doi: 10.1016/j.micres.2014.06.002

Bahar, O., Levi, N., and Burdman, S. (2011). The cucurbit pathogenic bacterium *Acidovorax citrulli* requires a polar flagellum for full virulence before and after host-tissue penetration. *Mol. Plant Microbe Interact.* 24**,** 1040-1050. doi: 10.1094/Mpmi-02-11-0041

Han, T., You, C., Zhang, L., Feng, C., Zhang, C., Wang, J., et al. (2016). Biocontrol potential of antagonist *Bacillus subtilis* Tpb55 against tobacco black shank. *BioControl* 61**,** 195-205.

Jain, R., and Pandey, A. (2016). A phenazine-1-carboxylic acid producing polyextremophilic *Pseudomonas chlororaphis* (MCC2693) strain, isolated from mountain ecosystem, possesses biocontrol and plant growth promotion abilities. *Microbiol. Res.* 190**,** 63-71. doi: 10.1016/j.micres.2016.04.017

Yuan, L.L., Li, Y.Q., Wang, Y., Zhang, X.H., and Xu, Y.Q. (2008). Optimization of critical medium components using response surface methodology for phenazine-1-carboxylic acid production by Pseudomonas sp M-18Q. *J. Biosci. Bioeng.* 105**,** 232-237. doi: 10.1263/jbb.105.232


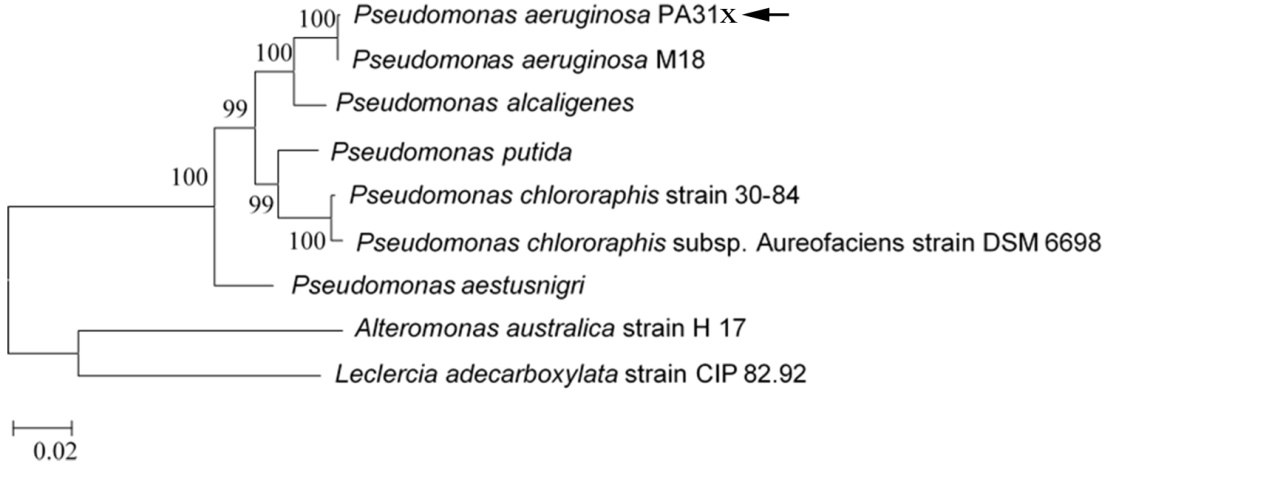


**FIGURE S1.** The consensus phylogenetic tree of *P. aeruginosa* PA31x with other related strains obtained from GenBank constructed by the neighbor-joining method. Numbers above the branches are bootstrap values based on 1000 replicates.


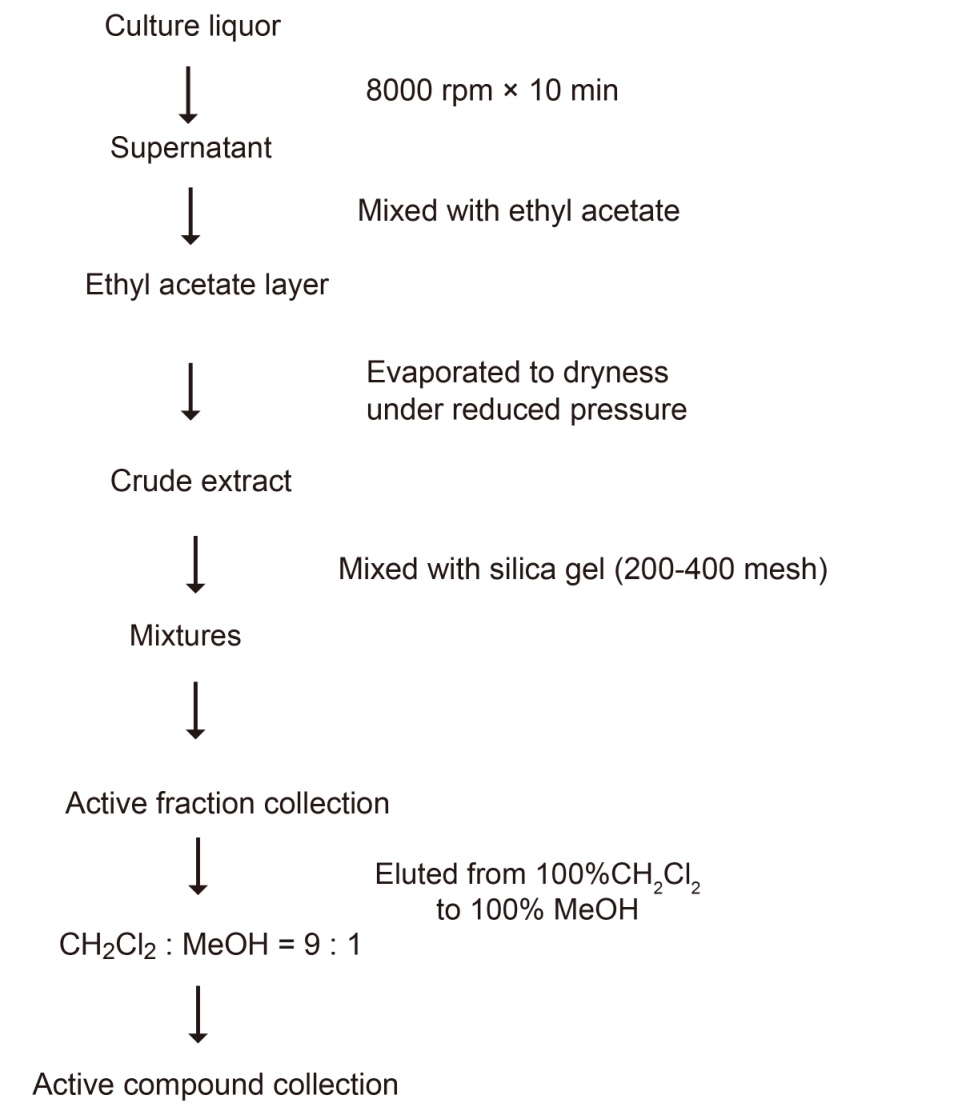


**Figure s2.** Purification scheme of the active compound produced by *P. aeruginosa* PA31x.

**
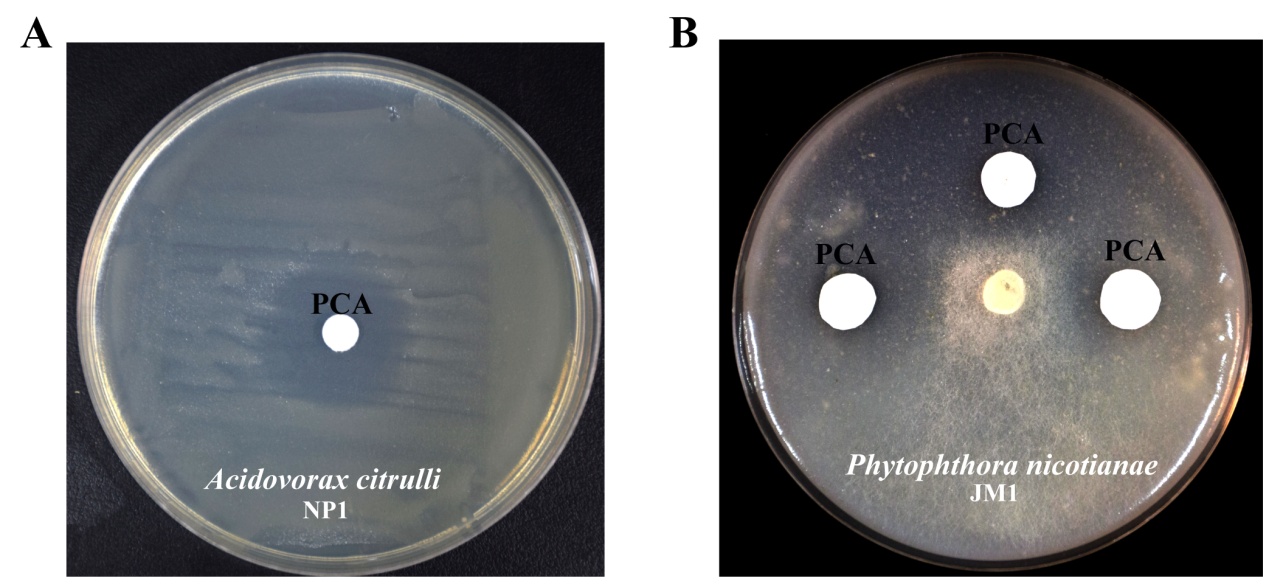
**

**Figure s3. The antimicrobial activity assays of purified PCA against plant pathogens *A. citrulli* NP1 (A) and *P. nicotianae* JM1 (B).** Sterile filter paper discs (diameter 10 mm) impregnated with PCA (the final concentration was 10 mg/mL) were applied in this assay.

**
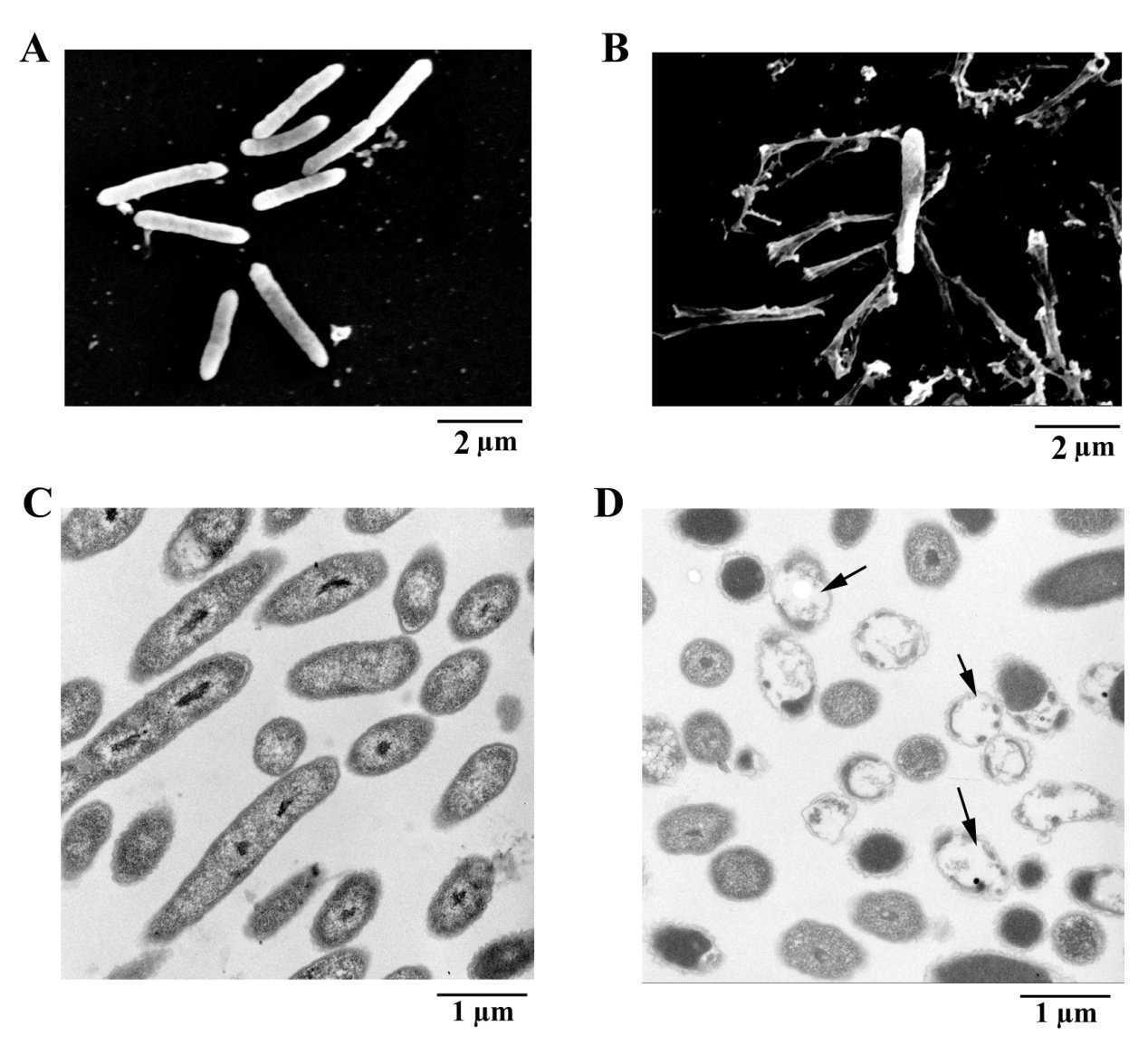
**

**Figure s4. Electron microscopic observation of morphological changes of *A. citrulli* NP1 cells following the treatment of PCA.** SEM images of *A. citrulli* NP1 cells without **(A)** or with the treatment of PCA **(B)**. TEM images of *A. citrulli* NP1 cells without **(C)** or with the treatment of PCA **(D)**.

**
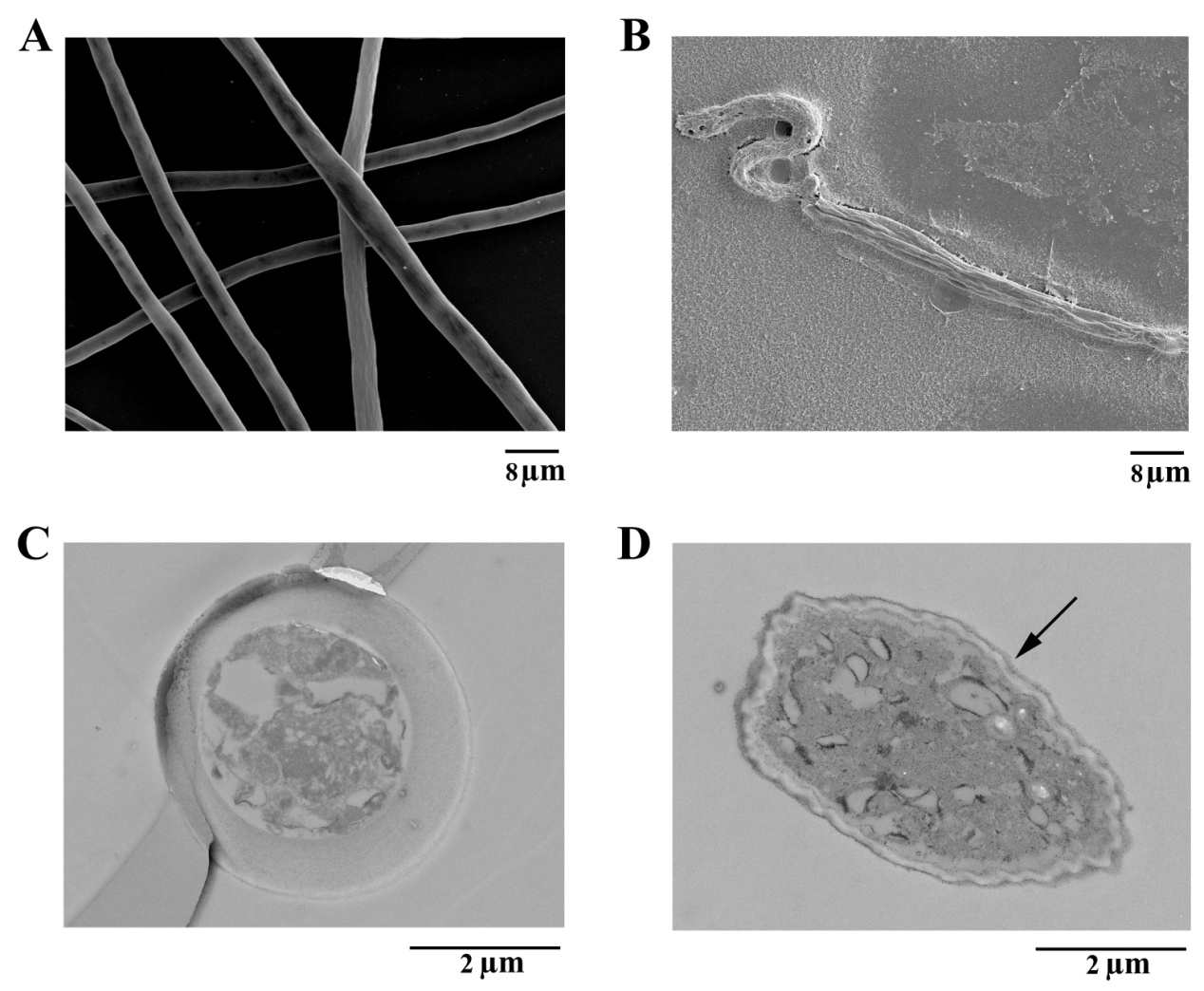
**

**Figure s5. Electron microscopic observation of morphological changes of *P. nicotianae* JM1 mycelia following the treatment of PCA.** SEM images of *P. nicotianae* JM1 mycelia without **(A)** or with the treatment of PCA **(B)**. TEM images of *P. nicotianae* JM1 mycelia without **(C)** or with the treatment of PCA **(D)**.

**
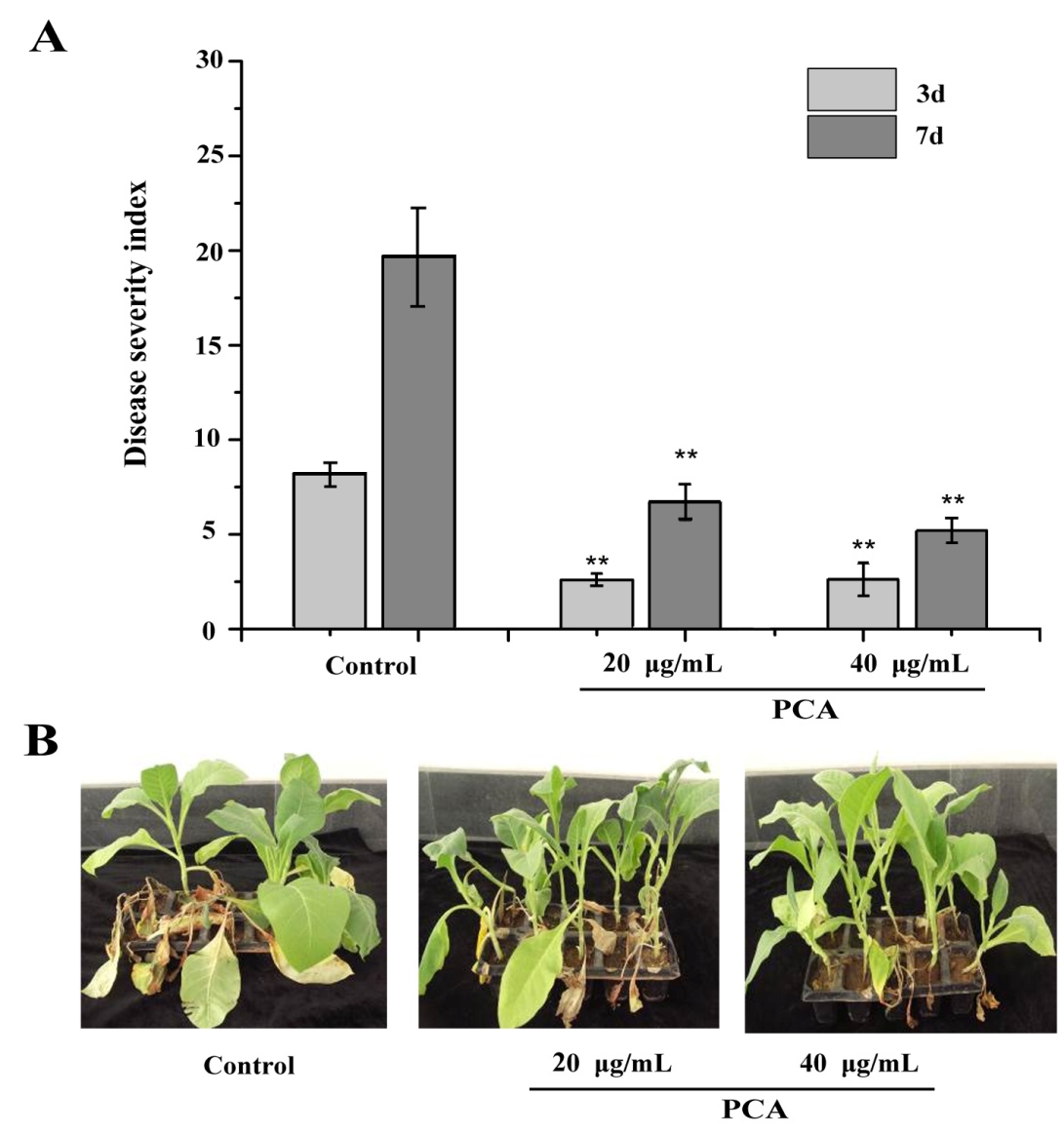
**

**Figure s6. Inhibitory activities of PCA against *P. nicotianae* JM1 infection to tobacco. (A)** Disease severity index on tobacco black shank**.** Error bars indicate the standard deviations of three measurements. ***P* < 0.01 versus the control. The error bars (SD) from the mean for three replicates are shown. (**B**) The typical disease severity pictures of tobacco infected by *P. nicotianae* JM1 in the absence or presence of different concentrations of PCA.

**TABLE S1. ^1^H Nuclear magnetic resonance (NMR) signals from the active compound isolated from *P. aeruginosa* PA31x**

| Hydrogen number | ^δ^H (ppm) |
| --- | --- |
| H-2 | 8.53 (dd) |
| H-3 | 8.34 (dd) |
| H-4 | 8.99 (dd) |
| H-6 | 8.28 (dd) |
| H-7 | 8.00-8.04 (m) |
| H-8 | 8.00-8.04 (m) |
| H-9 | 8.00-8.04 (m) |
| COOH | 15.55 (s) |

^δ^H:125 MHz, chemical shift in ppm.

Characters: s (singlet), dd: (double of doublets), m: (multiplet).

**TABLE S2. ^13^C Nuclear magnetic resonance (NMR) signals from the active compound isolated from *P. aeruginosa* PA31x**

| Carbon number | ^δ^C (ppm) |
| --- | --- |
| C-1 | 125.15 |
| C-2 | 130.39 |
| C-3 | 137.53 |
| C-4 | 135.21 |
| C-4a | 139.97 |
| C-5a | 140.18 |
| C-6 | 131.84 |
| C-7 | 128.10 |
| C-8 | 130.21 |
| C-9 | 133.32 |
| C-9a | 143.51 |
| C-10a | 144.21 |
| COOH | 160.00 |

^δ^C: 500 MHz, chemical shift in ppm.

**TABLE S3. The primers used for cloning of *phz1* gene cluster in this study.**

| Primers | Sequence of primers |
| --- | --- |
| phz1-1F | GAGGGAAAAGTTTCTCCGGCATAC |
| phz1-1R | CCAGAAATGATTGGGGTCGTC |
| phz1-2F | CCTGCGCCGCCATGAACTT |
| phz1-2R | TATACGCCGCACAGCACCAA |
| phz1-3F | CACGACATGCAGCGCTACTTCCTAC |
| phz1-3R | GATCAGGATCGCCGAGTCCAG |
| phz1-4F | TCGGTCCCTACCTCAAGGAGATGGC |
| phz1-4R | TGCGGTAGACCTCGATGGGAAAG |
| phz1-5F | TCTTCACCCCGGTCAACGAACTGCC |
| phz1-5R | CGATGGGTTCGCTCATGGGTGCTTC |

**TABLE S4. The primers used for cloning of *phz2* gene cluster in this study.**

| Primers | Sequence of primers |
| --- | --- |
| phz2-1F | CGGCAATTTCTCCGGCCTGTCATC |
| phz2-1R | AGCATCTCGTGGCTGGTCCAGAC |
| phz2-2F | GTACGCCAAGCCGCGTTCCAA |
| phz2-2R | CTTGCGGTTGTCGAGGAATTCCATG |
| phz2-3F | AGGGCGCCTACTGGACCTTCATC |
| phz2-3R | GGATCGGCTGCTGCATGCTG |
| phz2-4F | GGGAGATGAACCTGTCGGAGAGCAC |
| phz2-4R | GCGTGCGGTTGGATGGGTTC |

**TABLE S5. The primers used for cloning of *phzM,* *phzS* and *phzH* in this study.**

| Primers | Sequence of primers |
| --- | --- |
| phzM-F | CCTGGAGATCGCCAACGAACGCTAC |
| phzM-R | CGCTTTCCGTGGTCCAGTTGCC |
| phzS-F | GGAGTCGGTGGAGTTCTGGGGCAA |
| phzS-R | AGTGGAAGGGGCGGGCGGAAG |
| phzH-F | CGCACGGATGTTTTCAGCATGTTCT |
| phzH-R | CGAAATGCCCGACGCCTGAAGA |
